# Human foreskin fibroblast produces interleukin-6 to support derivation and self-renewal of mouse embryonic stem cells

**DOI:** 10.1186/scrt120

**Published:** 2012-07-31

**Authors:** Yu Ma, Junjie Gu, Chunliang Li, Xiaoyuan Wei, Fan Tang, Guilai Shi, Jing Jiang, Ying Kuang, Jinsong Li, Zhugang Wang, Xin Xie, Ying Jin

**Affiliations:** 1 Key Laboratory of Stem Cell Biology, Institute of Health Sciences, Shanghai Jiao Tong University School of Medicine & Shanghai Institutes for Biological Sciences, Chinese Academy of Sciences, 225 South Chongqing Road, Shanghai 200025, China; 2 Shanghai Stem Cell Institute, Shanghai Jiao Tong University School of Medicine, 225 South Chongqing Road, Shanghai 200025, China; 3 Department of Tumor Cell Biology, St. Jude Children's Research Hospital, 262 Danny Thomas Place, Memphis, TN 38103, USA; 4 Laboratory of Receptor-based BioMedicine, School of Life Sciences and Technology, Shanghai Key Laboratory of Signaling and Disease Research, Tongji University, Shanghai 200092, China; 5 Laboratory of Molecular Cell Biology, Institute of Biochemistry and Cell Biology, Shanghai Institute for Biological Sciences, Chinese Academy of Sciences, Shanghai 200031, China; 6 Shanghai Research Center for Model Organisms, Shanghai 201203, China; 7 State Key Laboratory of Drug Research, National Center for Drug Screening, Shanghai Institute of Materia Medica, Chinese Academy of Sciences, Shanghai 201203, China

## Abstract

**Introduction:**

Embryonic stem cells (ESCs) provide an attractive cell source for basic research and disease treatment. Currently, the common culture system for mouse ESC requires mouse embryonic fibroblast (MEF) as a feeder layer supplemented with leukemia inhibitory factor (LIF). The drawbacks associated with MEF and the cost of LIF have motivated exploration of new feeder cell types to maintain self-renewal of mouse ESCs without the need of exogenous LIF. However, why these feeder cells could maintain ESCs at the undifferentiated state independent of exogenous LIF is unclear.

**Methods:**

We derived mouse ESC lines using human foreskin fibroblast (HFF) in the absence of exogenous LIF. We also examined the dependence of HFF on the JAK-Stat3 pathway to maintain ESC identities and explored the potential molecular basis for HFF to support self-renewal of ESCs.

**Results:**

HFF supported mouse ESC self-renewal superiorly to MEFs. Using the HFF system, multiple lines of mouse ESCs were successfully derived without addition of exogenous LIF and any small molecular inhibitors. These ESCs had capacities to self-renew for a long period of time and to differentiate into various cell types of the three germ layers both *in vitro *and *in vivo*. Moreover, the ESCs participated in embryonic development and contributed to germ cell lineages in the chimeric mouse. At a molecular level, HFF was dependent on the JAK-Stat3 pathway to maintain ESC self-renewal. The high level of interleukin-6 (IL-6) produced by HFF might be responsible for the exogenous LIF-independent effect.

**Conclusion:**

This study describes an efficient, convenient and economic system to establish and maintain mouse ESC lines, and provides insights into the functional difference in the support of ESC culture between MEF and HFF.

## Introduction

Embryonic stem cells (ESCs) are derived from the inner cell mass of blastocyst [1]. These cells can proliferate indefinitely *in vitro *and differentiate into all of the cell types of the three embryonic germ layers (endoderm [2-4], mesoderm [5-7] and ectoderm [8,9]) as well as germ cells [10,11]. The unique properties of ESCs - unlimited self-renewal and pluripotency - hold great potential both in basic research and clinical applications. To maintain the properties of ESCs *in vitro*, the culture condition is extremely important. In early research, mouse embryonic fibroblast (MEF) feeder cells, serum and leukemia inhibitory factor (LIF) were utilized in mouse ESC culture. Later it was found that LIF collaborated with bone morphogenetic protein 4 (BMP4) could maintain the self-renewal of mouse ESCs in the absence of feeder cells and serum [12]. As one of the members of IL-6 family cytokines [13,14], LIF binds to the extracellular parts of transmembrane protein LIF receptor, leading to the formation of heterodimers of LIF receptor and gp130. The intracellular parts of the heterodimers recruit Janus kinase (JAK) and are activated sequentially. In the downstream, signal transducer and activator of transcription 3 (Stat3) in the cytoplasm is phosphorylated and forms homodimers, followed by their entering into the nucleus to activate the downstream genes required to maintain the self-renewal of mouse ESCs [15-18]. Stat3 is thus a critical component of the LIF-JAK-Stat3 pathway to sustain ESCs in an undifferentiated state.

The maintenance of mouse ESCs at a ground state of self-renewal in the absence of LIF and serum was recently reported using two inhibitors (2i) of fibroblast growth factor/extracellular signal-related kinase 1/2 and glycogen synthase kinase 3 [19]. Nevertheless, MEF and LIF are widely utilized for derivation and routine culture of mouse ESCs due to the fact that mouse ESCs self-renew better in the presence of both MEF and LIF. In particular, the efficiency of establishing mouse ESC lines in the presence of MEF and LIF is markedly higher than without them. MEF is generally prepared from embryos of E13.5 and used as feeder cells for ESC derivation or culture after the inactivation using mitomycin C or gamma radiation. MEF provides the essential matrix and some anti-differentiation factors, including LIF, to support the self-renewal of ESCs [13,14]. However, LIF produced by MEF is not enough to maintain ESC properties most of the time. As a result, exogenous recombinant LIF is often added to the culture. Although mouse ESCs grow well under the culture conditions containing both LIF and MEF, several drawbacks exist: the recombinant LIF is expensive; only the early passages of MEF could be used to support ESC culture, leading to the need to make MEF frequently [20,21]; frequent preparation of MEF results in batch to batch variations as well as possible contaminations of pathogens; and the ability of MEF to support the ESC culture lasts for only a short time after gamma radiation or mitomycin C treatment.

These disadvantages associated with the culture system using MEF and LIF have greatly limited the *in vitro *large-scale expansion of ESCs. Exploring an effective, convenient and inexpensive culture system for ESC culture is therefore necessary. In fact, cells from other species or tissues, such as human foreskin fibroblast (HFF) [22,23], human amnion epithelial cells [24], human endothelial cell line [25] and rabbit spleen fibroblast-like cells [26], have been used in the ESC culture. In these culture systems, exogenous LIF was not needed and feeder cells could proliferate *in vitro *for a long period of time. A recent study reported derivation of mouse ESCs on HFF in the presence of 2i and adrenocorticotropic hormone fragments 1-24 [23]. Nevertheless, whether these alternative feeder cells could support the derivation of mouse ESCs in the absence of LIF or inhibitors remains unclear, as does the underlying mechanism by which the feeder cells maintain the self-renewal of mouse ESCs without the need for exogenous LIF.

In this study, we established several mouse ESC lines of the C57BL/6 strain using HFF in the absence of exogenous LIF and inhibitors. Additionally, we analyzed the cytokines produced by HFF to investigate how HFF supported mouse ESC derivation and culture at the molecular level. To our best knowledge, this is the first report of generating mouse ESC lines on human feeder cells without addition of LIF and inhibitors. The culture system established in this study is effective, convenient and inexpensive for both routine and large-scale culture of mouse ESCs.

## Materials and methods

### Derivation and culture of mouse embryonic stem cells

All animal procedures were performed according to the guidelines approved by the Shanghai Jiao Tong University School of Medicine (20080050). E3.5 mouse blastocysts of the C57BL/6 strain were flushed from the mouse uterus and plated onto HFF that had been inactivated by mitomycin C or gamma radiation. The undifferentiated outgrowths were mechanically split 4 or 5 days later and plated onto inactivated HFF. After an additional 3 days, the undifferentiated parts of the colonies were picked up and digested by 0.05% trypsin/EDTA (Gibco, Grand island, NY, USA) into single cells for passaging. The derivation and culture medium was DMEM (Gibco) with 10% fetal bovine serum, 100 U/ml penicillin, 100 μg/ml streptomycin, 0.1 mM β-mercaptoethanol (Sigma, St. Louis, MO, USA) and 2 mM L-glutamine.

To determine the effect of the JAK pathway, 10 or 20 μM JAK inhibitor (Calbiochem, Darmstadt, Bundesland, Germany) was added to the cell culture to inhibit the phosphorylation of Stat3.

### Alkaline phosphatase staining

ESC colonies were fixed with 4% paraformaldehyde and permeabilized by 0.1% Triton X-100 in PBS. The Alkaline Phosphatase Substrate Kit III (Vector Laboratories, Inc., Burlingame, CA, USA) was used for staining at 37°C for 15 minutes.

### Immunofluorescence staining

Immunofluorescence staining was performed as described previously [27]. Supplier information for antibodies used in this study is provided in Table 1.

**Table 1:** Suppliers of antibodies

Antibody	Company
Oct4	Prepared in our laboratory
Sox2	Prepared in our laboratory
Nanog	Santa Cruz (Santa Cruz, CA, USA)
SSEA1	Chemicon (Billerica, MA, USA)
SSEA4	Chemicon
Gata4	Santa Cruz
Cdx2	Santa Cruz
Map2	Santa Cruz
Flk1	Santa Cruz
Foxa2	Abcam ({Cambridge, MA, USA)
Vimentin	DAKO (Carpinteria, CA, USA)
Tuj1	Promega (Madison, WI, USA)

### Teratoma formation

About 5 × 10^6 ^ESCs were collected and injected intramuscularly into SCID-beige mice. About 4 to 6 weeks later, teratomas were harvested and sectioned for H & E staining.

### Blastocyst injection and embryo transfer

The procedures of blastocyst injection and embryo transfer were described previously [28]. E14 mouse ESCs were from 129 mice and injected into the blastocysts of C57BL/6 mice. C57H1.2 mouse ESCs were injected into blastocysts of ICR mice.

### Germline participation detection

C57H1.2 ESCs were transfected with EGFP-PPY vector and selected by puromycin for 3 days. EGFP-labeled ESCs were injected into blastocysts for generation of chimeric embryos. Genital ridges of the chimeric embryos at E15.5 were obtained and sliced. Immunofluorescence staining was performed on these sections with antibodies against EGFP and Oct4.

### RT-PCR and quantitative RT-PCR

Total RNA of mouse ESCs was extracted by TRIzol (Invitrogen, Carlsbad, CA, USA). Four micrograms of RNA was reverse-transcribed into cDNA using oligod(T)16 and ReverTra Ace reverse transcriptase (Toyobo, Osaka, Japan). PCR was carried out in a 20 μl system comprised of 1 μl cDNA, 250 nM each primer pair, 200 μM dNTP and 1 U Taq DNA polymerase in a thermocycler. The primers used were reported previously [28] with 26 reaction cycles for *Oct4*, *Sox2 *and *Nanog *and 21 reaction cycles for *Gapdh*, respectively. The quantitative RT-PCR analysis was carried out according to the procedures of the manufacture on an ABI PRISM 7900 (Applied Biosystems, Foster City, CA, USA) with fluorogenic SYBR Green double-stranded DNA-binding dye (Applied Biosystems). Primers were designed inside the homologous region of mouse and human *LIF *with the forward sequence 5'-ACTGGCACAGCTCAATGGC-3' and the reverse sequence 5'-GATCTTCTGGTCCCGGGTG-3'.

### Spontaneous differentiation of mouse embryonic stem cells

Mouse ESCs were suspended in low-attached dishes for 3 days to form embryoid bodies (EBs). These EBs were then attached to the gelatin-coated slides for an additional 3 days and fixed with 4% paraformaldehyde for immunofluorescence staining.

### Combined bisulfite restriction analysis

Genomic DNA (200 ng) was restricted with EcoRV (Takara, Dalian, Liaoning Province, China) and treated with sodium bisulfite as previously described [29]. Treated DNA was subjected to the nested PCR analysis. PCR products were restricted with TaqI (Takara), followed by electrophoresis in a 2% agarose gel.

### Western blotting

Cell lysate of mouse ESCs in Co-IP buffer containing 10 mM Hepes (pH 7.6), 250 mM sodium chloride, 0.1% Nonidet P-40, 5 mM EDTA, 1 mM phenylmethanesulfonyl fluoride and 1 mM sodium fluoride was collected and quantified by the BCA kit (Pierce, Rockford, IL, USA). Fifteen micrograms of proteins in supernatant with 4× loading buffer in a total volume of 24 μl were boiled at 100°C to denature the proteins. The denatured proteins were resolved by SDS-PAGE and transferred to nitrocellulose membranes (Schleicher & Schuell, Keene, NH, USA). The membrane was blocked by 5% skim milk in Tris-buffered saline with Tween-20 and incubated with antibodies against total Stat3 (Cell Signaling Technology, Beverly, MA, USA), pStat3 (Cell Signaling Technology) or α-tubulin (Sigma) overnight at 4°C. The membrane was washed three times with Tris-buffered saline containing Tween-20 and was incubated in peroxidase-conjugated secondary antibodies for 2 hours at room temperature. Western blotting detection kits (Pierce; and Millipore, Billerica, MA, USA) were used to detect the specific protein bands.

### ELISA

MEF and HFF were treated with mitomycin C and 2 × 10^5 ^treated cells were replated onto 6 cm cell culture dishes. Three milliliters of the ESC culture medium were conditioned for 24 hours. The conditioned medium was collected and the concentration of IL-6 was detected by mouse IL-6 and human IL-6 Quantikine ELISA kits (R&D, Minneapolis, MN, USA), respectively.

### Cytokine array assay

Mouse ESC medium without LIF was conditioned by MEF and HFF, respectively, for 24 hours and collected for cytokine array assay using the RayBio^® ^Human Cytokine Antibody Array kit (RayBiotech, Norcoss, GA, USA). The assay was conducted as instructed by the manufacturer. The intensities of signals were quantified by densitometry. The cytokines having markedly higher levels in the HFF-conditioned medium than in the MEF-conditioned medium are shown in Table 2.

**Table 2:** Cytokine array results

Cytokine	MEF conditioned medium (control)	HFF conditioned medium	HFF conditioned medium/MEF conditioned medium
Angiogenin	0.131048401	0.456743843	3.485306508
CNTF	0.261190473	0.420619379	1.610393268
EGF	0.239486275	0.463340184	1.934725419
Eotaxin	0.081703409	0.273209001	3.343911894
GM-CSF	0.054976951	0.098757079	1.796339322
IGFBP-2	0.469018849	0.936722625	1.997196118
IGFBP-4	0.17012308	0.330308218	1.941583814
IL-6	0.055000597	0.258231969	4.695075723
IL-17	0.030446833	0.071105402	2.335395632
MCP-1	0.322883585	0.584756604	1.811044694

CNTF, ciliary neurotrophic factor; EGF, epidermal growth factor; GM-CSF, granulocyte-macrophage colony-stimulating factor; HFF, human foreskin fibroblast; IGFBP, insulin-like growth factor binding protein; MCP, monocyte chemotactic protein; MEF, mouse embryonic fibroblast.

### Statistical analysis

All values were analyzed by Student's *t *test or one-way ANOVA to determine the significance of the differences. *P *< 0.05 was considered statistically significant.

## Results

### HFF maintains mouse ESC properties in the absence of exogenous LIF

**Figure 1:**
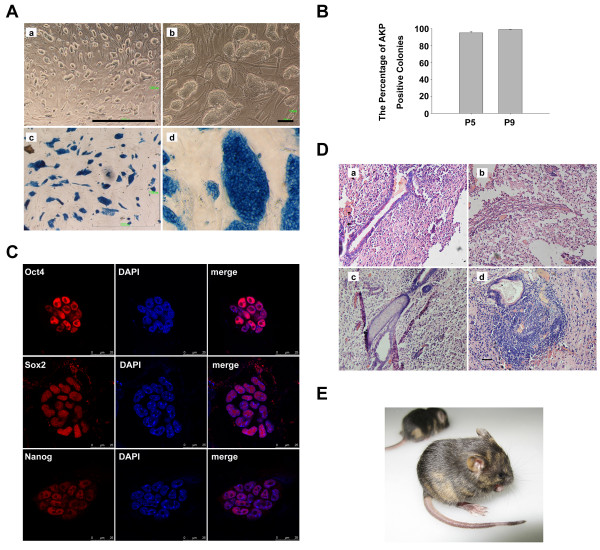
**Characterization of mouse embryonic stem cells cultured on human foreskin fibroblast without exogenous leukemia inhibitory factor**. **(A) **Morphology of E14 mouse embryonic stem cells (ESCs) cultured on human foreskin fibroblast (HFF) without exogenous leukemia inhibitory factor (LIF) for 20 passages. **(a), (b) **Bright field of E14 mouse ESCs on HFF. **(c), (d) **Alkaline phosphatase (AKP) staining of E14 mouse ESCs on HFF. Scale bars: (a), (c) 1,000 μm; (b), (d) 200 μm. **(B) **Quantitative analysis of the AKP-positive colonies of E14 mouse ESCs cultured on HFF without exogenous LIF at passages 5 and 9, respectively; *n *= 3. **(C) **Immunofluorescence staining of E14 mouse ESCs on HFF without exogenous LIF using antibodies against Oct4, Sox2 and Nanog, respectively. Scale bars: 25 μm. 4',6-Diamidino-2-phenylindole (blue) staining was used to indicate the nucleus. **(D) **H & E staining of the teratoma formed by E14 mouse ESCs cultured on HFF without exogenous LIF for 16 passages. The teratoma contains **(a) **intestinal epithelium, **(b) **smooth muscles, **(c) **cartilages and **(d) **neural epithelium. Scale bars: 50 μm. **(E) **Chimeric mice obtained post injection of E14 mouse ESCs cultured on HFF without exogenous LIF for 16 passages into blastocysts of C57BL/6 mice. Brown color denotes origination of the E14 cells.

Our previous study demonstrated that HFF cells generated in our own laboratory could proliferate for more than 20 passages *in vitro *and efficiently support the derivation of mouse induced pluripotent stem cells without exogenous LIF [28]. To further verify the role of HFF in the maintenance of ESC properties, we cultured E14 mouse ESCs on HFF without exogenous LIF. After more than 10 passages, ESCs sustained an undifferentiated morphology incompacted colonies, displaying a high nucleus-to-cytoplasm ratio, prominent nucleolus and obvious boundary on the margin of the colonies (Figure 1A,a,b). The alkaline phosphatase (AKP) staining, indicating an undifferentiated status, was positive (Figure 1A,c,d,B). The ESCs expressed pluripotency-associated markers such as Oct4, Sox2 and Nanog, as determined by immunofluorescence staining (Figure 1C). These observations indicated that HFF was able to sustain mouse ESCs at the self-renewal state independent of exogenous LIF.

We next determined the ability of HFF to maintain the pluripotency of ESCs. E14 mouse ESCs cultured on HFF without exogenous LIF for 16 passages were injected intramuscularly into the SCID-beige mice to test their ability to form teratomas, which is one of the standard tests for the pluripotent developmental potential of ESCs *in vivo*. Teratomas formed about 4 weeks after injection. The H & E staining results showed that the teratomas contained cells and tissues originated from three germ layers, such as intestinal epithelium (endoderm), smooth muscles (mesoderm), cartilages (mesoderm) and neural epithelium (ectoderm) (Figure 1D). Moreover, we tested whether these cells could participate in the embryonic development of the mice by injecting E14 mouse ESCs, which had been cultured on HFF without exogenous LIF for 16 passages, into the blastocysts of C57BL/6 mice. Chimeric offsprings were obtained (Figure 1E). This finding clearly demonstrated that ESCs maintained on HFF without exogenous LIF were developmentally pluripotent.

### HFF sustains ESCs at an undifferentiated state superiorly to MEF in the absence of exogenous LIF

**Figure 2:**
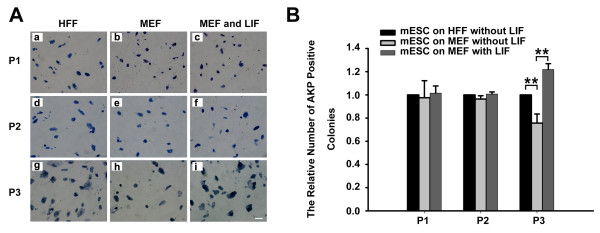
**Human foreskin fibroblast supports self-renewal of mouse embryonic stem cells better than mouse embryonic fibroblast**. **(A) **Alkaline phosphatase (AKP) staining of E14 mouse embryonic stem cells (ESCs) cultured on human foreskin fibroblast (HFF), mouse embryonic fibroblast (MEF) without exogenous leukemia inhibitory factor (LIF) or MEF with LIF for one, two and three passages, respectively. Scale bars: 100 μm. **(B) **The quantitative analysis of the results in (A). AKP-positive colonies in 10 (100×) microscopic fields were counted. The number of the AKP-positive colonies on HFF is set as 1.0. **P < 0.01, *n *= 3.

To further characterize the ability of HFF to support the self-renewal of mouse ESCs, we compared the ability of HFF to maintain ESCs in an undifferentiated state with that of MEF without exogenous LIF. For the first and second passages, AKP staining showed no significant difference between ESCs cultured on HFF and those on MEF. However, substantially fewer AKP-positive colonies were detected for cells grown on MEF, as compared with those on HFF, when the cells were passaged three times (Figure 2A). Statistical analysis showed that AKP-positive colonies accounted for only 70% of the ESCs on MEF compared with AKP-positive colonies on HFF (Figure 2B). This finding is consistent with the fact that MEF alone is not sufficient to maintain mouse ESCs in a self-renewal state and with the fact that supplemental LIF is required for routine mouse ESC culture. However, HFF possesses the ability to maintain ESC properties without the need for exogenous LIF for a long period of time.

### Mouse ESC lines can be successfully generated on HFF without exogenous LIF

**Figure 3:**
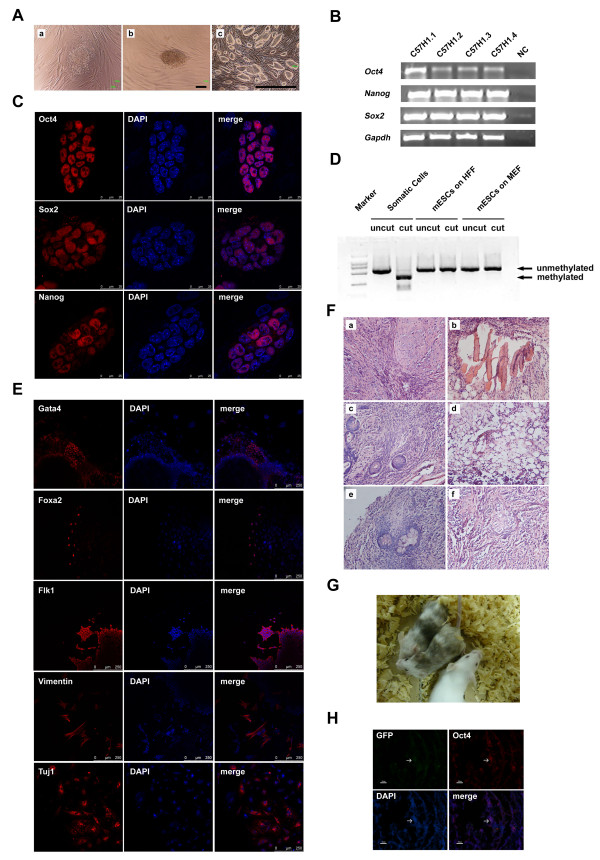
**Derivation and characterization of mouse embryonic stem cells on human foreskin fibroblast without exogenous leukemia inhibitory factor**. **(A) **Derivation of C57H1.2 mouse embryonic stem cells (ESCs) on human foreskin fibroblast (HFF) without exogenous leukemia inhibitory factor (LIF). **(a) **Outgrowth formed post inner cell mass (ICM) attachment. **(b) **Manually split ICM clumps. **(c) **Typical mouse ESC colonies. Scale bars: (a), (b) 100 μm; (c) 1,000 μm. **(B) **RT-PCR analysis of the expression levels of the pluripotency-associated markers (*Oct4*, *Nanog *and *Sox2*) in newly derived mouse ESC lines on HFF without exogenous LIF. NC, water was used as the negative control. **(C) **Immunostaining of C57H1.2 mouse ESCs with antibodies against Oct4, Sox2 and Nanog. Scale bars: 25 μm. **(D) **Detection of the methylation status at the endogenous *Oct4 *promoter of C57H1.2 mouse ESCs by a combined bisulfite restriction analysis. **(E) **Immunofluorescence staining of the attached embryonic bodies formed by C57H1.2 mouse ESCs with antibodies against Gata4, Foxa2, Flk1, Vimentin and Tuj1. Scale bars: 250 μm. **(F) **Teratomas formed by C57H1.2 mouse ESCs were sectioned and stained with H & E. Typical **(a, b) **smooth muscles, **(c, e) **intestinal epithelium, **(d) **fat and **(f) **neural-like tissues were found. Scale bars: 50 μm. **(G) **Chimeric mice generated by injection of C57H1.2 mouse ESCs into blastocysts of the ICR mouse. Black colors indicate origination of C57H1.2 mouse ESCs. **(H) **Immunofluorescence staining of the genital ridge sections from a chimeric embryo (male) at E15.5 with antibodies against EGFP and Oct4. C57H1.2 ESCs were labeled with EGFP. Arrow, an EGFP and Oct4 double-positive cell. Scale bars: 50 μm.

Given that derivation of ESC lines requires more optimal conditions than the maintenance of established ESC lines, we sought to determine whether, in the absence of exogenous LIF, new mouse ESC lines could be derived on HFF. To this end, E3.5 blastocysts of C57BL/6 mice, which are considered more difficult than 129 mice in terms of the generation of ESC lines [30], were plated onto HFF without exogenous LIF (Figure 3A,a). A few days later, the undifferentiated cell clusters in the outgrowth were picked up manually and transferred onto the fresh HFF (Figure 3A,b). After two rounds of mechanical splitting, the undifferentiated ESC colonies on HFF were passaged enzymatically using trypsin-EDTA. Typical undifferentiated mouse ESC colonies could be observed easily (Figure 3A,c). In total, we derived four mouse ESC lines on HFF - named C57H1.1, C57H1.2, C57H1.3 and C57H1.4, respectively. Semiquantitative RT-PCR analysis showed that cells from these four lines expressed pluripotency-associated markers, *Oct4*, *Sox2 *and *Nanog *(Figure 3B). Among the four cell lines, C57H1.2 has been cultured on HFF without exogenous LIF for more than 50 passages. We further characterized this line in the following experiments.

Immunofluorescence staining results illustrated the nuclear localization of proteins of Oct4, Sox2, and Nanog in the ESCs of the C57H1.2 line (Figure 3C). Moreover, the combined bisulfite restriction analysis showed the unmethylated promoter status of the *Oct4 *gene, further validating that these ESCs were in the undifferentiated state (Figure 3D).

To determine the differentiation potential of the newly derived C57H1.2 ESCs, we cultured ESCs in suspension for EB formation. After 3 days of suspension culture, the EBs were plated onto gelatin-coated dishes for an additional 3 days. Immunofluorescence staining results showed that EB-derived differentiated cells expressed Gata4, Foxa2 (endoderm), Flk1, Vimentin (mesoderm) and Tuj1 (ectoderm), suggesting that C57H1.2 ESCs possessed the capacity to generate a variety of cell types from three embryonic germ layers *in vitro *(Figure 3E). In addition, C57H1.2 ESCs formed teratomas 4 to 6 weeks after they were intramuscularly injected into SCID-beige mice. The H & E staining of teratoma sections detected the tissues and cells derived from the three germ layers, including the smooth muscle, intestinal epithelium, fat, and neural-like tissues (Figure 3F). C57H1.2 ESCs were thus capable of differentiating into various cell types or tissues of three germ layers *in vivo *as well. As chimera formation is a more stringent test to evaluate the pluripotency of mouse ESCs, we injected C57H1.2 ESCs into blastocysts of ICR mice. The generation of the chimeric offspring with mosaic black coat colors in a high proportion demonstrated their abilities to participate in the embryonic development of the mouse (Figure 3G). Furthermore, we assessed the germline transmission of C57H1.2 ESCs, which were stably labeled with EGFP before blastocyst injection. Genital ridges of chimeric embryos at E15.5 were collected and their sections were stained with antibodies against EGFP and Oct4. Although the efficiency was very low, we found EGFP and Oct4 double-positive cells in the genital ridge of a male embryo, implying the participation of the C57H1.2 ESCs to germline development (Figure 3H). Collectively, our data supported the conclusion that HFF could support the derivation of pluripotent mouse ESCs in the absence of exogenous LIF and any other inhibitors.

### Activation of Stat3 is essential for the self-renewal of mouse ESCs on HFF

**Figure 4:**
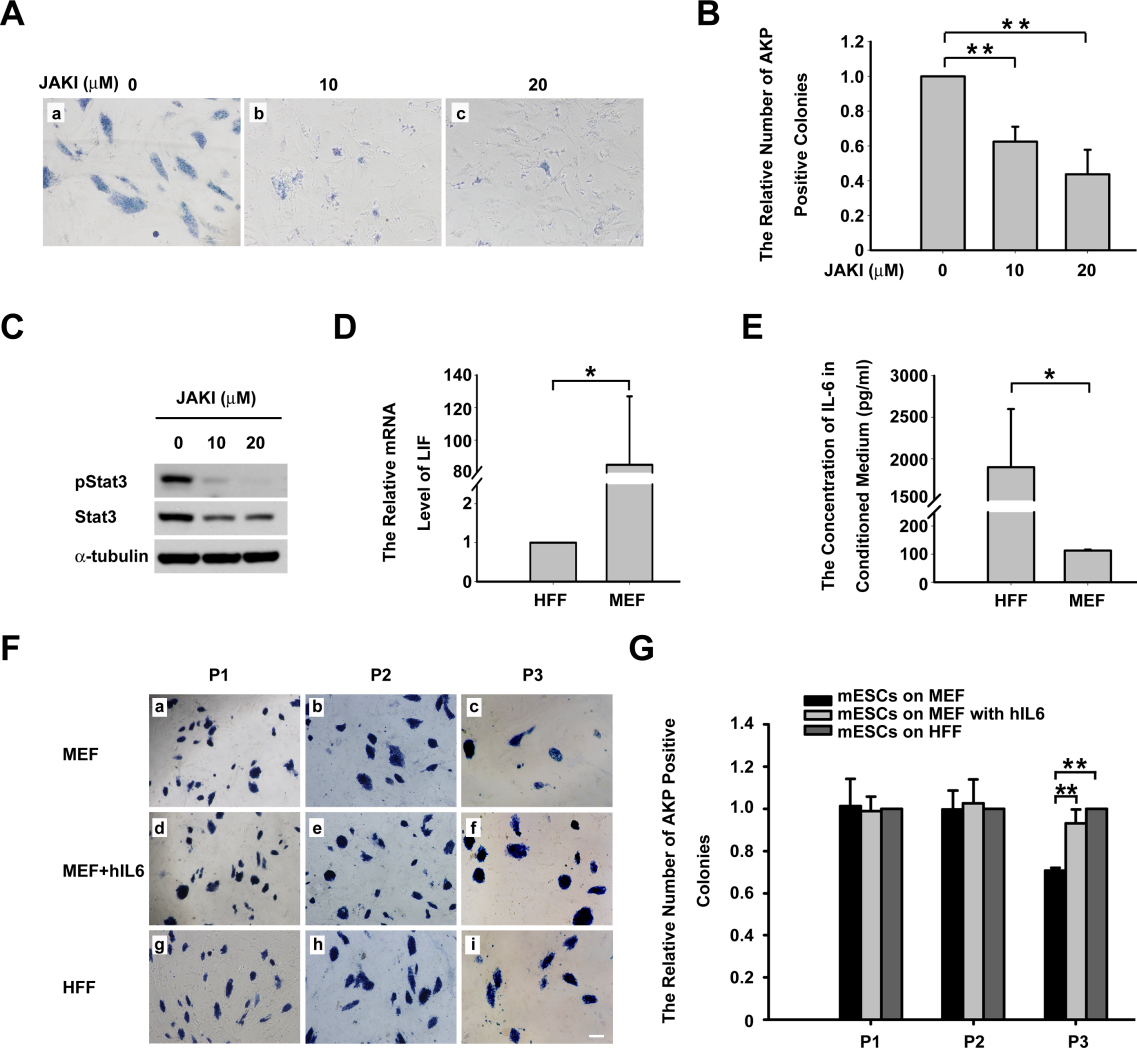
**Human foreskin fibroblast supports self-renewal of mouse embryonic stem cells through the JAK-Stat3 pathway**. **(A) **Alkaline phosphatase (AKP) staining of C57H1.2 mouse embryonic stem cells (ESCs) cultured on human foreskin fibroblast (HFF) treated with **(a) **0 μM (dimethylsulfoxide (DMSO) only or **(b) **10 μM and **(c) **20 μM JAK inhibitor (JAKI). **(B) **Quantitative analysis of AKP-positive colonies of C57H1.2 mouse ESCs cultured on HFF with 0 μM (DMSO only), 10 μM and 20 μM JAKI. AKP-positive colonies in 10 (100×) microscopic fields were counted. The number of AKP-positive ESC colonies cultured with 0 μM JAKI is set as 1.0. Scale bars: 50 μm. **P < 0.01, *n *= 3. **(C) **Western blot analysis of the levels of phosphorylated Stat3 in C57H1.2 mouse ESCs grown on HFF treated with 0 μM (DMSO only), 10 μM and 20 μM JAKI, respectively. Experiments were conducted three times and the representative result is shown. **(D) **Quantitative RT-PCR analysis of the relative expression level of LIF in MEF and HFF. *P < 0.05, *n *= 3. The mRNA level of LIF in HFF is set as 1. **(E) **Comparison of the concentration of secreted IL-6 by ELISA. *P < 0.05, *n *= 3. **(F) **AKP staining of C57H1.2 mouse ESC colonies cultured on MEF alone, or MEF with 20 ng/ml human IL-6 (hIL-6) or HFF alone for one, two and three passages, respectively. Scale bars: 100 μm. **(G) **Quantitative analysis of results in (F). AKP-positive colonies in 10 (100×) microscopic fields were counted. The number of AKP-positive colonies of C57H1.2 mouse ESCs (mESCs) on HFF is set as 1.0. **P < 0.01, *n *= 3.

It is known that the JAK-Stat3 pathway is essential for the self-renewal of mouse ESCs when they grow on MEF. We have here demonstrated that mouse ESCs could self-renew for a long period of time on HFF without exogenous LIF, but it also became necessary to determine whether the self-renewal of ESCs on HFF was also dependent on the JAK-Stat3 pathway. Since phosphorylation of Stat3 is the key step for JAK-Stat3 signaling to support the self-renewal of mouse ESCs and JAK is responsible for the phosphorylation of Stat3, we examined whether the JAK inhibitor affected the self-renewal of ESCs cultured on HFF. Morphologically, the colonies of inhibitor-treated ESCs were smaller than those in the control group (vehicle only) (Figure 4A). Moreover, inhibitor treatment reduced the number of AKP-positive colonies significantly (Figure 4B). Of note, these effects of the JAK inhibitor were dosage dependent. The specific effect of the JAK inhibitor on the level of phosphorylated Stat3 was validated by western blot analysis. As expected, the inhibitor reduced the level of phosphorylated Stat3 in a dosage-dependent manner, demonstrating an efficient block of the JAK activity by the inhibitor (Figure 4C). These observations suggest that the activation of Stat3 is essential for the supportive effect of HFF on the self-renewal of mouse ESCs.

Given that both MEF and HFF were dependent on the JAK-Stat3 pathway to maintain ESCs in an undifferentiated state, why did MEF, but not HFF, require exogenous LIF? Because human LIF can bind to LIF receptors on the membrane of mouse ESCs and activate the downstream LIF-JAK-Stat3 pathway [18], we suspected that HFF might produce more LIF than MEF. However, we found that the transcript level of *LIF *in HFF was significantly lower than that in MEF as determined by quantitative RT-PCR assays using primers flanking the homologous region of the human and mouse *LIF *(Figure 4D), which negated our hypothesis.

To investigate which other factors secreted by HFF might be associated with its supportive effect on the self-renewal of mouse ESCs, we carried out a cytokine screen assay with the conditioned medium collected from either MEF or HFF. Interestingly, the assay detected a 3.5-fold higher level of IL-6 in the HFF-conditioned medium than in MEF-conditioned medium (Table 2). To verify this difference, we conducted the ELISA assay. A 15-fold higher level of IL-6 was found in HFF-conditioned medium, as compared with MEF (Figure 4E). The discrepancy in the fold of difference in the IL-6 level between HFF and MEF found by the two assays could result from the different detection sensitivity associated with individual assays. To further verify the roles of IL-6 for the self-renewal of mouse ESCs, C57H1.2 mouse ESCs were cultured on MEF supplemented with 20 ng/ml recombinant human IL-6 for three passages. Compared with ESCs cultured on MEF without LIF and exogenous IL-6, the addition of human IL-6 significantly increased the number of AKP-positive colonies to a level comparable to that on HFF (Figure 4F,G). This finding clearly showed that IL-6 was sufficient to maintain mouse ESC self-renewal on MEF. As IL-6 is in the same cytokine family as LIF and can also activate the phosphorylation of Stat3 through gp130 and JAK [28], it is reasonable to deduce that HFF-produced IL-6 is the major player in the function of HFF to sustain the self-renewal of mouse ESCs.

## Discussion

The drawbacks of MEF and the cost of LIF have motivated us to explore a more convenient, efficient and costless ESC culture system. Here, we have demonstrated that HFF supported mouse ESC self-renewal superiorly to MEF and that the activation of Stat3 was required for the HFF to act as functional feeder cells. Importantly, mouse ESCs having long-term self-renewal ability and full developmental potential were generated without the need of exogenous LIF and any small molecular inhibitors. This study therefore reports a robust and cost-effective cell culture system for both establishment and routine culture of mouse ESCs. As the feeder cells are of human origin, this system could potentially be applied to the derivation and culture of human ESCs under xeno-free conditions.

The advantage of HFF over MEF for ESC culture and derivation is obvious. HFF cells are so stable that they display homogeneous morphology even after they are passaged more than 20 times. This property makes it unnecessary to make feeder cells frequently and prevents the variance among different batches. Moreover, HFFs are more durable than MEFs in that they remain in healthy condition more than 2 weeks after inactivation by radiation. In contrast, MEF deteriorates within 1 week after the inactivation. Furthermore, for experiments associated with detection of gene expression in mouse ESCs, usage of HFF as feeder cells enables one to design species-specific detections, precluding the possible contamination of the feeder cells. The usage of HFF for ESC culture is hence superior to MEF due to the convenience. In addition, the HFF culture system is more economic, since MEF, but not HFF, as feeder cells for mouse ESC culture needs exogenous LIF. Last, but not least, another benefit of using HFF as the feeder is its potential in the establishment and maintenance of xeno-free human pluripotent stem cell lines. MEF expresses nonhuman sialic acid Neu5Gc, which may lead to immune-reactivity *in vivo *when human ESCs cultured on MEF are used for transplantation [31]. Therefore, compared with the traditional MEF culture system, the HFF system is indeed more convenient, economic and efficient. The HFF system can be widely applied in the large-scale expansion of the ESCs *in vitro*. Human ESCs were previously reported to be successfully derived on human feeders [32,33]. HFF can therefore be used as feeder cells in the human ESC culture to eliminate contaminations of animal origin.

Why HFF, but not MEF, sustains ESC self-renewal without exogenous LIF has remained elusive. Our finding that inhibition of JAK prevents HFF from maintaining the ESC self-renewal argues for a key role of the JAK-Stat3 signaling pathway in the control of the self-renewal of ESCs cultured on HFF. Hence, similar to MEF, HFF supported the maintenance of ESC properties through the JAK-Stat3 pathway. Different from MEF, however, HFF secreted a high level of IL-6 to activate the pathway. LIF belongs to a family of cytokines, which includes IL-6, ciliary neurotrophic factor, IL-11 and oncostatin M[34]. Interestingly, our cytokine screen and ELISA assays found a substantially higher level of IL-6 in the HFF-conditioned medium than in the MEF-conditioned medium. In addition, several other cytokines were found to have higher levels in the HFF-conditioned medium, including ciliary neurotrophic factor. We therefore speculate that the higher concentration of IL-6 and related cytokines produced by HFF could explain its unique property to sustain the self-renewal of ESCs independent of exogenous LIF. Furthermore, it was reported that factors such as basic fibroblast growth factor secreted by HFF might be involved in the self-renewal of mouse ESCs [35]. We therefore do not exclude the possibility that other factors produced by HFF also play a role in HFF-supported mouse ESC self-renewal.

## Conclusion

In summary, this study demonstrates that HFF system is convenient and economic to efficiently derive and maintain mouse ESC lines. The identification of IL-6 as a primary cytokine produced by HFF provides insights into the differences in support of mouse ESC cultures between MEF and HFF.

## Abbreviations

AKP: alkaline phosphatase; DMEM: Dulbecco's modified Eagle's medium; E: embryonic day; EB: embryonic body; ELISA: enzyme-linked immunosorbent assay; ESC: embryonic stem cell; GFP: green fluorescent protein; H & E: hematoxylin and eosin; HFF: human foreskin fibroblast; IL: interleukin; JAK: Janus kinase; LIF: leukemia inhibitory factor; MEF: mouse embryonic fibroblast; PBS: phosphate-buffered saline; PCR: polymerase chain reaction; RT: reverse transcription; Stat3: signal transducer and activator of transcription 3.

## Competing interests

The authors declare that they have no competing interests.

## Authors' contributions

YM was responsible for designing the study, characterization of ESCs, cell culture, testing of the Stat3-dependent pathway and manuscript writing. JG was responsible for cell culture and establishment of ESC lines, MEF preparation, and animal-associated experiments. CL was responsible for designing of the study, cell culture and establishment and characterization of the ESC lines. XW, YK, ZW and XX were responsible for acquisition of the chimeric mice from C57H1.2 ESCs. FT and GS were responsible for characterization of ESCs. JJ and JL were responsible for acquisition of the chimeric mice from E14 ESCs. YJ was responsible for designing the study, data analysis, manuscript writing as well as providing financial support and final approval of the manuscript. All authors read and approved the final manuscript.
